# Universal cervical‐length screening to prevent preterm birth in twin pregnancy: cost‐utility analysis

**DOI:** 10.1002/uog.29287

**Published:** 2025-07-10

**Authors:** Y. Khaikin, R. A. Gladstone, K. E. Murphy, N. Melamed, P. Pechlivanoglou

**Affiliations:** ^1^ Department of Obstetrics & Gynaecology University of Toronto Toronto ON Canada; ^2^ Institute of Health Policy, Management, and Evaluation University of Toronto Toronto ON Canada; ^3^ Division of Maternal Fetal Medicine Mount Sinai Hospital Toronto ON Canada; ^4^ Division of Maternal Fetal Medicine Sunnybrook Health Sciences Centre Toronto ON Canada; ^5^ Child Health Evaluative Sciences The Hospital for Sick Children Toronto ON Canada

**Keywords:** cervical‐length measurement, cost‐utility analysis, premature birth, twin pregnancy

## Abstract

**Objective:**

The purpose of this cost‐utility analysis was to model the clinical and economic impact of three cervical‐length screening strategies among low‐risk twin pregnancies: two‐step universal screening (at 18–20 and 20–22 weeks), one‐step universal screening (at 18–20 weeks) and no screening.

**Methods:**

This study used a decision‐analytic model (decision tree and cohort state transition model) with a 100‐year time horizon in a Canadian context. The population included dichorionic diamniotic twin pregnancies without a history of preterm birth or prophylactic progesterone or cerclage. The model assumed that vaginal progesterone was initiated for cervical length ≤ 25 mm and that cervical cerclage was performed plus vaginal progesterone treatment for cervical length ≤ 15mm. The primary outcomes were total lifetime health‐related costs (in 2023 Canadian dollars ($)), quality‐adjusted life years (QALYs) and incremental cost‐effectiveness ratios. Clinical outcomes included the probability of preterm birth (≤ 28 and ≤ 34 weeks), probability of stillbirth and life expectancy. Probabilistic and deterministic sensitivity analyses were carried out.

**Results:**

Base‐case and probabilistic sensitivity analysis showed that, when compared with no screening, the two‐step screening strategy increased the QALYs modestly (0.62 (95% credible interval (CrI), −0.16 to 1.41)) and decreased lifetime costs (−$2460 (95% CrI, −$4850 to $251)) by reducing the rate of preterm birth. The one‐step screening strategy, although inferior to the two‐step screening strategy, also increased the QALYs and reduced costs. Findings consistent with these were obtained on testing of the model assumptions with deterministic sensitivity analysis.

**Conclusions:**

This cost‐utility analysis supports a universal two‐step screening strategy for twin pregnancies in a Canadian context. Although the conclusions of this analysis are robust in terms of the sensitivity analysis, more reliable predictions of long‐term costs and quality of life require more twin‐specific lifetime data. Additionally, cost‐utility analyses in other healthcare contexts are needed. © 2025 The Author(s). *Ultrasound in Obstetrics & Gynecology* published by John Wiley & Sons Ltd on behalf of International Society of Ultrasound in Obstetrics and Gynecology.

## INTRODUCTION

Preterm birth (PTB) is the leading cause of neonatal morbidity and mortality among twins^1^, ^2^, ^3^, ^4^. Although twins account for just 3.2% of all births, they contribute 20% of PTBs^5^, ^6^. The economic burden of PTB is significant owing to the costs of neonatal intensive care^7^, long‐term health and education expenses, and indirect effects on parental productivity^8^, ^9^. Early detection of twin pregnancies at risk for PTB can facilitate preventive intervention.

Cervical‐length (CL) measurement by transvaginal ultrasound during the second trimester is a predictor of PTB^10^, ^11^, ^12^. Evidence‐based interventions exist to reduce the risk of PTB when CL is short. A meta‐analysis found that among twin pregnancies with CL ≤ 25 mm, vaginal progesterone decreased the risk of birth < 33 weeks^13^, while universal prophylactic progesterone treatment has been shown not to affect the risk of PTB^14^. When CL is ≤ 15 mm, cervical cerclage placement may reduce the risk of spontaneous PTB^15^. There is a need for a coordinated, systematic approach to the identification of twin pregnancies at increased risk of PTB, which may benefit from preventive intervention.

Although CL screening can identify patients at increased risk of PTB, universal screening protocols present a burden to the healthcare system and increase the frequency of treatment and associated costs. Studies investigating the cost‐effectiveness of screening strategies among singletons have found that universal CL screening of low‐risk pregnancies is cost‐effective^16^, ^17^, ^18^, ^19^, ^20^. A Swedish cost‐effectiveness analysis^16^ modeled four screening approaches, ranging from universal screening to screening only high‐risk pregnancies, and found that all four screening strategies improved health outcomes compared with no screening.

There have been no decision‐analytic evaluations of universal CL screening for twin pregnancies. Furthermore, the optimal screening approach for short cervix in twin pregnancies is unknown, with some evidence suggesting that serial measurement, rather than a single visit, improves the detection of a short cervix^11^. Given varying recommendations from professional societies regarding CL screening for twin pregnancies^21^, cost‐utility data could help to inform policymakers regarding the best strategies.

The objective of this study was to model long‐term cost‐utility outcomes for three CL screening strategies among low‐risk twin pregnancies: two‐step universal screening (at 18–20 and 20–22 weeks), one‐step universal screening (at 18–20 weeks) and no screening.

## METHODS

This study was reported in accordance with the Consolidated Health Economic Evaluation Reporting Standards (CHEERS 2022) checklist^22^. We designed a cost‐utility analysis that relied on a decision‐analytic model^23^, the purpose of which was to compare the clinical and economic outcomes of three screening strategies: (1) no CL screening, (2) universal one‐step CL screening at 18–20 weeks' gestation and (3) universal two‐step CL screening at 18–20 and 20–22 weeks.

The model included dichorionic diamniotic twin pregnancies without additional risk factors for PTB. Pregnancies with a history of PTB and those already receiving either prophylactic progesterone or cervical cerclage were excluded. Monochorionic twin and higher‐order pregnancies were also excluded. We assumed that all pregnancies undergoing CL screening reached fetal viability, meaning that pregnancies resulting in miscarriage were excluded from the model.

Model outcomes were quality‐adjusted life years (QALYs), total lifetime health‐related costs (in 2023 Canadian dollars ($)) and incremental cost‐effectiveness ratios (ICERs). We report screening‐ and treatment‐related costs, as well as costs of birth and neonatal intensive care unit (NICU) admission. Clinical outcomes included the probability of PTB (≤ 28 and ≤ 34 weeks), probability of stillbirth and life expectancy.

### Model structure

**Figure 1:**
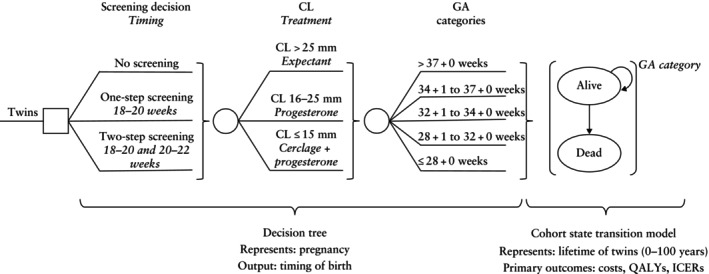
Simplified diagram of decision‐analytic model for comparing cervical‐length (CL) screening strategies to prevent preterm birth in twin pregnancy. The square node represents the clinical decision and circular nodes represent possible outcomes. Patients in the no‐screening strategy group do not receive cervical‐length based treatment. GA, gestational age at birth; ICERs, incremental cost‐effectiveness ratios; QALYs, quality‐adjusted life years.

The model contained two components (Figure 1): a decision tree^24^ modeling pregnancy and birth outcomes, and a cohort state transition model (cSTM)^25^ representing the lifetime of the twins from birth to a maximum of 100 years, with a 1‐year cycle length.

Patients not undergoing CL screening and those with CL > 25 mm were managed expectantly. Patients who underwent universal screening were assumed to receive interventions based on their CL measurement as follows: (1) for CL ≤ 25 mm, vaginal progesterone once daily from diagnosis of short cervix to 36 gestational weeks; (2) for CL ≤ 15 mm, ultrasound‐indicated cervical cerclage plus vaginal progesterone once daily from diagnosis of short cervix to 36 weeks. In the two‐step strategy, treatment for short cervix was determined based on the shortest CL measurement from the two ultrasound assessments. The model assumed that CL never increased with subsequent measurement because a patient's risk of PTB and continuation of progesterone therapy is unlikely to be altered by a longer measurement. The disutility of both progesterone and cerclage treatment is likely negligible compared to the disutility of PTB and was not included in the model.

The outcome of the decision tree was timing of birth. For each strategy, the probability of birth was distributed across five gestational‐age (GA) categories from ≤ 28 to > 37 weeks. Birth‐timing probabilities were used to determine NICU admission costs and to populate parameters for the cSTM. These categories were chosen because of clinical relevance and to accommodate available data.

The cSTM followed newborn twins from age 0 to a maximum of 100 years and contained two states: alive, including alive with morbidity, and dead, the absorbing state. The model was generated for each of the five GA categories, incorporating annual health‐related quality of life (HRQoL) and costs. The GA category‐specific outcomes were then summed with weights based on the birth‐time distribution, resulting in the overall strategy outcomes. We assumed that each pregnancy resulted in two neonates with identical outcomes. In accordance with the Canadian Guidelines for the Economic Evaluation of Health Technologies^26^, a 1.5% discount rate was used in the base case. These assumptions were tested in scenario analyses.

Model verification occurred in several iterations. We designed the model in keeping with existing work among singletons^16^, ^18^, ^20^. The model structure and input parameters were agreed on by all team members, including an expert in twin pregnancies (N.M.), an expert in decision analysis (P.P.) and two senior obstetrics resident physicians (Y.K. and R.A.G.).

A short list of model parameters and their sources, clinical ranges and probability distributions is given in Table 1. A summary of data sources (Table S1), the full list of model parameters (Table S2) and detailed calculations (Appendix S1) are also given.

**Table 1:** Decision‐tree probabilities for modeling twin pregnancy and birth outcomes

Model parameter	Base case	Clinical range	Probability distribution
Cervical‐length screening detection*			
Screen one CL ≤ 25 mm	0.044	0.03–0.06	beta(32, 696)
Screen one CL ≤ 15 mm, given CL ≤ 25 mm	0.468	0.31–0.64	beta(15, 17)
Screen two CL ≤ 25 mm, given screen one negative (> 25 mm)	0.076	0.06–0.10	beta(53, 643)
Screen two CL ≤ 15 mm, given CL ≤ 25 mm and screen one negative	0.377	0.26–0.51	beta (20, 33)
Progesterone effect (RR) on probability of birth**†** at GA:			
≤ 28 + 0 weeks	0.53	0.24–1.16	lognormal(log(0.53), 0.40)
28 + 1 to 32 + 0 weeks	0.71	0.34–1.47	lognormal(log(0.71), 0.37)
32 + 1 to 34 + 0 weeks	0.64	0.31–1.33	lognormal(log(0.64), 0.37)
34 + 1 to 37 + 0 weeks	1.02	0.72–1.43	lognormal(log(1.02), 0.17)
Cerclage effect**‡** (RR) on probability of birth**§** at GA:			
≤ 28 + 0 weeks	0.56	0.26–1.21	lognormal(log(0.56), 0.39)
28 + 1 to 32 + 0 weeks	0.78	0.38–1.60	lognormal(log(0.78), 0.37)
32 + 1 to 34 + 0 weeks	0.3	0.11–0.84	lognormal(log(0.30), 0.53)
34 + 1 to 37 + 0 weeks	1	0.60–1.67	lognormal(log(1.00), 0.26)

Probability distributions are presented as beta (number of events, number of non‐events) or lognormal(log(mean), standard error).

Full list of model parameters is available in Table S2.

* Source: Melamed *et al*. (2016)^27^.

† Source: Romero *et al*. (2022)^13^.

‡ Effect of cerclage in combination with progesterone is unknown and assumed conservatively to be same as effect of cerclage alone.

§ Source: Roman *et al*. (2015)^15^.

CL, cervical length; GA, gestational age; RR, relative risk.

### Probabilities

Twin‐specific probabilities for CL screening outcomes and birth at each GA category, depending on the CL, were determined from previously published retrospective observational studies^11^, ^27^. These studies included data from 441 patients with a twin pregnancy at a tertiary care center, in which all patients had serial measurements of CL every 2–3 weeks, starting from 18 to 22 weeks. CL measurements were used to predict the probability of PTB. The efficacy of progesterone (relative risk (RR)) in reducing PTB probability was calculated from an individual‐patient data meta‐analysis (six trials, *n* = 303)^13^, and the efficacy of cerclage (without concurrent progesterone) was sourced from a multicenter retrospective cohort study (*n* = 140)^15^. Because data on the effect of cerclage combined with progesterone were not available, we assumed conservatively that the effect of cerclage plus progesterone was equivalent to that of cerclage alone. The model assumed that all patients underwent treatment based on their CL, and this assumption was tested in a scenario analysis. Probabilities of stillbirth at different GAs at birth were sourced from a meta‐analysis of dichorionic twin pregnancies (25 studies, *n* = 29 685)^28^.

### Annual mortality rate

Annual all‐cause mortality rates were sourced from the latest available Canadian lifetable data (2020) via the Human Mortality Database^29^. These values were assigned to the term birth category. This decision assumed that twins born at term had the same mortality risk compared with the general population; there is evidence to support this assumption, both in the neonatal period and for the entire lifespan^30^, ^31^. GA‐category‐specific mortality rates were then calculated by multiplying the annual mortality rate at term by birth‐timing‐specific and age‐specific adjusted hazard ratios up to the latest available age of 45 years. These data are available from a Swedish national cohort study of singleton livebirths between 1973 and 2015^32^. For ages 46–99 years, we assumed the hazard of death to be the same across GA categories.

### Annual health‐related quality of life

Annual HRQoL values were calculated for each GA category from 0 to 100 years using several steps. The details of our approach, as well as its assumptions and limitations, are described in Appendix S2. In brief, a population trend was identified via Health Utility Index Mark 3 (HUI3) data collected in the latest available Canadian Community Health Survey (2015–2016, *n* = 2769)^33^. Because of a paucity of long‐term data for PTB specifically, HUI3 estimates for these subgroups were sourced from a prospective Canadian study of neonates born with extremely low birth weight (< 1000 g) with or without neurosensory impairment (NSI)^34^. The proportion of NSI for each GA category was sourced separately from two observational studies^35^, ^36^. Our approach did not account for the effect of a different mortality rate in the group with NSI on the overall HUI3 averages.

### Costs

We assumed that patients having at least one scan as part of the CL screening protocol would have subsequent weekly scans up to 24 weeks as part of surveillance. The cost of progesterone was calculated per week, assuming a dose of 200 mg vaginally daily, taken from 18 to 36 weeks or birth, whichever came sooner^37^. The cost of birth was a weighted sum of a typical vaginal birth and a Cesarean section, assuming a Cesarean section rate of 60%^38^, ^39^. This value was varied in the sensitivity analysis to include the full range of possible birth/NICU admission costs. NICU admission rates were assumed to be 100% for twins born ≤ 34 + 0 weeks. Admission rates for late preterm and term neonates were sourced from a Californian population‐based cohort study of births between 2010 and 2018 (*n* = 320 340)^40^. These NICU admission rates were similar to the rates at our tertiary care center.

Annual costs from age 0 to 9 years were sourced from a model incorporating administrative data in Quebec, Canada^8^. This model included costs associated with resource utilization, direct medical care, parental out‐of‐pocket expenses and education. Costs after 9 years are unknown and were assumed to be equal to the average cost at age 9 years across all GA categories. Although costs are reported to decline with each additional year since PTB^8^, healthcare costs in general increase in later life^41^ and our assumption likely underestimated lifetime costs. All costs were adjusted to 2023 Canadian dollars using the Bank of Canada inflation calculator^42^ and all postbirth costs were doubled to account for both twins.

### Sensitivity analyses

Deterministic sensitivity analysis (DSA) was used to test assumptions in the model. For the DSA, either one (one‐way) or two (two‐way) model parameters were changed with each run of the model, while keeping the other parameters fixed. The parameters were set to plausible clinical ranges, with most representing 95% CIs from data sources. For each scenario, the strategy with the greatest net benefit was determined. One‐way DSA was used for total costs, QALYs and probability of PTB (≤ 28 and ≤ 34 weeks). Two‐way analyses for costs were used for progesterone effectiveness *vs* cost and cerclage effectiveness *vs* cost. Scenario analysis included major assumptions of the model: discounting, treatment uptake rates and a multiplier for twin outcomes, which was varied from 1 (i.e. singleton outcome) to 2 (perfect concordance between twins and the assumption of the model). Probabilistic sensitivity analysis (PSA) was used to reflect uncertainty in base‐case parameters. Each model parameter was sampled from its probability distribution over 1000 simulations. The outcomes were reported as means with 95% credible intervals (CrIs), along with ICER plots and a cost‐effectiveness acceptability curve. The model was created and analyzed using R (version 4.3.2; https://www.R‐project.org/). The full model code is available online at github.com/yannayk/CLS_twins_Rfiles.

## RESULTS

### Base‐case analysis

The two‐step screening strategy was dominant, with the lowest costs, highest QALYs and lowest rates of PTB ≤ 34 + 0 weeks, ≤ 32 + 0 weeks and ≤ 28 + 0 weeks, followed by the one‐step and no‐screening strategies (Table S3). Despite higher pregnancy‐related costs, the two‐step screening was cost‐saving overall because of the lower rates of PTB across all categories below 34 weeks. Stillbirth rates were higher for the two‐step screening strategy compared with one‐step screening and no screening (5.81, 5.77 and 5.74 per 1000 pregnancies, respectively).

**Figure 2:**
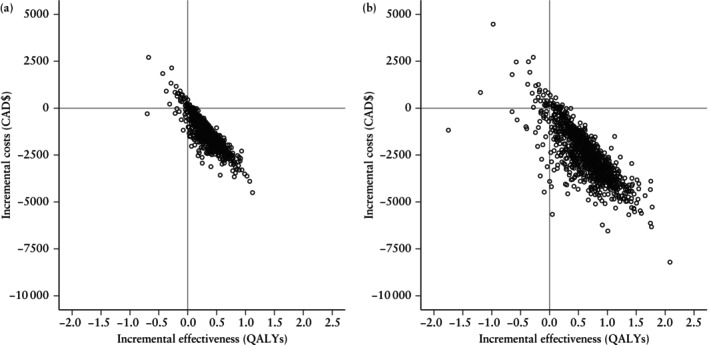
Probabilistic sensitivity analysis incremental cost‐effectiveness ratio plots comparing cervical‐length screening strategies to prevent preterm birth in twin pregnancy: (a) one‐step screening *vs* no screening and (b) two‐step screening *vs* no screening. Each point represents a single simulation (*n* = 1000). CAD$, 2023 Canadian dollars; QALYs, quality‐adjusted life years.

### Probabilistic sensitivity analysis

The outcomes of the PSA were similar to those of the base‐case analysis (Table 2). When the two screening strategies were compared with no screening, we found incremental gains in QALYs at lower costs (Figure 2). However, these benefits were small with high variability, particularly for the two‐step strategy. When compared with no screening, the ICERs were −$6553/QALY (95% CrI, −$12 921 to $2433) for two‐step screening and −$3943/QALY (95% CrI, −$12 031 to −$51) for one‐step screening.

**Table 2:** Outcomes of probabilistic sensitivity analysis comparing cervical‐length screening strategies to prevent preterm birth in twin pregnancy

Outcome	No screen	One‐step screening	Two‐step screening
Proportion screen positive with:			
CL ≤ 15 mm	0 (0–0)	0.02 (0.01–0.03)	0.05 (0.04–0.06)
CL ≤ 25 mm	0 (0–0)	0.04 (0.03–0.06)	0.12 (0.10–0.14)
Proportion preterm birth at:			
≤ 28 + 0 weeks	0.04 (0.02–0.05)	0.03 (0.02–0.04)	0.02 (0.01–0.04)
≤ 32 + 0 weeks	0.07 (0.06–0.09)	0.07 (0.05–0.09)	0.06 (0.04–0.08)
≤ 34 + 0 weeks	0.13 (0.11–0.16)	0.13 (0.10–0.15)	0.12 (0.10–0.14)
Stillbirths/1000 pregnancies	5.80 (4.67–7.06)	5.83 (4.70–7.10)	5.86 (4.70–7.18)
Life expectancy (years)	154.64 (152.85–156.18)	155.25 (153.47–156.75)	155.8 (154.04–157.37)
Cost per pregnancy (CAD$)			
Ultrasound	0 (0–0)	95 (89–102)	194 (186–203)
Progesterone	0 (0–0)	14 (10–19)	39 (32–46)
Cerclage	0 (0–0)	74 (42–116)	173 (128–226)
NICU admission	23 995 (20 807–27 361)	22 626 (19 640–26 196)	21 285 (18 299–25 090)
Total costs (CAD$)	46 133 (40 766–53 207)	44 871 (39 622–51 571)	43 672 (38 254–50 667)
Total QALYs	74.9 (54.2–86.0)	75.2 (54.4–86.4)	75.5 (54.4–87.0)
Incremental (*vs* no screen)			
Incremental QALYs	—	0.32 (−0.05 to 0.77)	0.62 (−0.16 to 1.41)
Incremental costs (CAD$)	—	−1262 (−2779 to 197)	−2460 (−4850 to 251)
ICER (CAD$/QALY)	—	−3943 (−12 031 to −51)	−6553 (−12 921 to 2433)

All values are reported as mean and 95% credible interval (*n* = 1000 simulations).

All costs represent 2023 Canadian dollars (CAD$).

Outcomes regarding twins are per pregnancy, assuming identical twin outcomes.

CL, cervical length; ICER, incremental cost‐effectiveness ratio; NICU, neonatal intensive care unit; QALY, quality‐adjusted life years.

**Figure 3:**
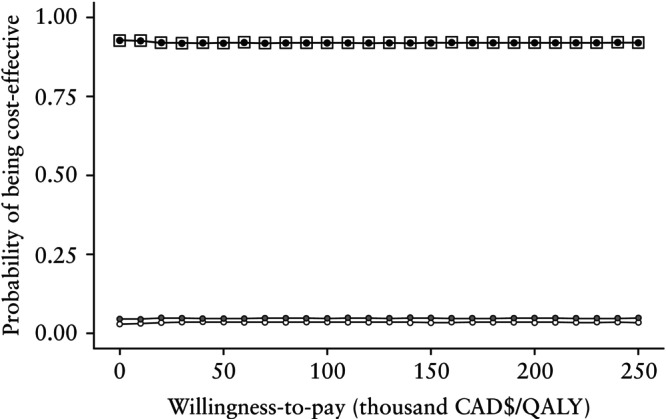
Cost‐effectiveness acceptability curves (1000 simulations) for three cervical‐length screening strategies to prevent preterm birth in twin pregnancy: 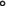, no screening; 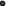, one‐step screening; and 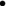, two‐step screening. 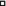, efficiency frontier; CAD$, 2023 Canadian dollars; QALY, quality‐adjusted life year.

The cost‐effectiveness acceptability curve indicates, for each strategy, the probability that the intervention is cost‐effective, given an increasing willingness to pay for each additional QALY gained (Figure 3)^43^. The two‐step screening strategy was dominant at all willingness‐to‐pay values, with a probability of being cost‐effective of 92–93%. In other words, given the data, there was a 92% chance that the additional cost of the two‐step screening strategy would be at or below an arbitrary ceiling of $100 000 per QALY gained.

### Deterministic sensitivity analysis

In total, 101 model parameters were included in the one‐way sensitivity analysis (Figure S1). Two‐step screening was the dominant strategy in the majority (> 99%) of scenarios. The dominant strategy only differed for two kinds of variable: NICU costs and cerclage plus progesterone effectiveness. When NICU costs for late PTB increased or those for extreme PTB (≤ 28 + 0 weeks) decreased, the two‐step screening strategy lost dominance in costs (Figure S1a). When NICU costs for infants delivered late preterm rose above $354 426, one‐step screening was dominant. Conversely, when NICU costs for infants born ≤ 28 + 0 weeks fell below $2781, no screening was the dominant cost‐minimizing strategy. The model was sensitive to the effectiveness of cerclage plus progesterone treatment in reducing the probability of extreme PTB. The no‐screening strategy minimized extreme PTB when the treatment RR of birth in the extreme PTB category (compared with no treatment) exceeded 1.17, and it minimized total costs when this treatment RR exceeded 1.15. Because of the effect of progesterone therapy alone, the model found a cost advantage for two‐step screening even when cerclage increased the RR of birth in the extreme PTB category.

For the remainder of the one‐way sensitivity analysis, two‐step screening was dominant for QALYs and costs across all ranges. Scenario analysis, including assumptions of the model (multiplying by 2 for twin outcomes, discounting and treatment uptake rates), did not change the model outcomes.

Two‐way sensitivity analysis was carried out for two types of parameter: progesterone effectiveness *vs* cost and cerclage effectiveness *vs* cost (Figure S2). Similar to the one‐way analysis, two‐step screening was dominant across most combinations. When cerclage plus progesterone treatment increased the probability of extreme PTB (RR > 1.17), no screening became the dominant strategy at any treatment cost. The two‐way analysis showed that when the cost of cerclage exceeded $3085, the treatment threshold was lower at RR > 1.15.

## DISCUSSION

In this cost‐utility analysis, we demonstrated that universal CL screening in twin pregnancies, with the purpose of reducing the rate of PTB and improving neonatal outcome, is cost‐effective compared with no screening. The two‐step screening strategy, with CL measurement at 18–20 and 20–22 weeks, showed dominance over the one‐step screening strategy (CL measurement at 18–20 weeks only) and no screening in terms of PTB rates, as well as lifetime costs and QALYs. Stillbirth rates were marginally higher in the screening strategy groups compared with no screening, a finding that is explained by the higher rates of stillbirth at term input into the model^28^. PSA and DSA demonstrated robust conclusions, with few exceptions. The model was sensitive to the relative NICU costs, as well as to the effectiveness of cerclage plus progesterone treatment. Notably, the differences between screening strategies in costs and QALYs were small. This may be because of several factors: the relatively small proportion of patients detected by screening, the fact that not all patients with short CL would benefit from treatment or the lack of data for lifetime costs and HRQoL.

To our knowledge, this is the first cost‐utility analysis of universal CL screening in twin pregnancy. Several decision analyses have been published on CL screening in singleton pregnancy^16^, ^17^, ^18^, ^19^, ^20^. With the exception of one study^17^, they conclude that CL screening in low‐risk populations is likely to be cost‐effective. Our analysis, although targeting a different population, reached a similar conclusion.

Because of the lack of available data, our model cannot predict reliably the long‐term health‐related costs associated with twin PTB. However, we have demonstrated that a universal screening strategy (either one‐step or two‐step) would be cost‐effective for twin pregnancies in the Canadian healthcare system. The proposed screening strategies could be integrated into existing antenatal care pathways. In fact, the Canadian Society of Obstetrics and Gynecology already recommends routine CL measurement for twin pregnancy^44^.

In developing the model, significant assumptions were made in light of missing information. Although our model was able to include twin data in the decision tree, there was a paucity of data specific to twin newborns and their lifespan in the cSTM. We suspect that populating more long‐term data in the cSTM would further penalize PTB outcomes and bolster the current conclusions. More evidence on the effect of cerclage at CL ≤ 15 mm is also needed, both with and without concurrent progesterone treatment. As more data become available, an update to this model's parameters or a microsimulation approach^45^ could be used to predict long‐term outcomes more accurately.

In addition to the lack of data, our approach also had other shortcomings. Our model is an approximation of the true system and we omitted some contributors to expected costs, quality of life and life expectancy. These included costs associated with establishing and implementing a new screening program (e.g. sterilization of ultrasound probes and longer examination duration), which were included in another model for CL screening among singletons^16^. Regarding external validation, the modeled PTB rate (< 37 + 0 weeks) for the no‐screening strategy (62.7%) was higher than the reported rate among twins in Canada (56.1%)^46^. This may be explained by our sourcing of birth‐timing probabilities from a tertiary care obstetric center, coupled with policies regarding the timing of birth of uncomplicated twin pregnancies.

There are several advantages to our model. Our model design involved five distinct GA categories, which allowed for inclusion of GA‐specific parameters. This might explain why our model, unlike previous analyses^17^, was insensitive to change in progesterone effectiveness. If progesterone is ineffective at reducing PTB in one GA category, it may still have a net benefit because of its effect in another. Our model also included cerclage as a treatment for short CL, which is not included in models of singletons^16^, ^17^, ^18^, ^20^.

In conclusion, this cost‐utility analysis of universal CL screening in twin pregnancy supports a routine two‐step CL screening strategy in a Canadian context. More reliable predictions of long‐term costs and QALYs require more twin‐specific lifetime data. Additionally, cost‐utility analyses in other healthcare models are needed.

## Supporting information


**Appendix S1** Detailed calculations (Excel spreadsheet)

**Appendix S2** Detailed explanation of annual lifetime Health Utility Index Mark 3 (HUI3) calculations
**Appendix S3** References for supplementary materials
**Table S1** Data sources
**Table S2** Complete model parameters
**Table S3** Model outcomes for the base case
**Figure S1** One‐way deterministic sensitivity analysis.
**Figure S2** Two‐way deterministic sensitivity analysis.

## Data Availability

The data that supports the findings of this study are available in the supplementary material of this article.
