# Supplementary material for: Universal cervical‐length screening to prevent preterm birth in twin pregnancy: cost‐utility analysis
Source: Ultrasound Obstet Gynecol. 2025 Jul 10;66(3):290–7. doi: 10.1002/uog.29287 (PMC12401501; doi:10.1002/uog.29287)
Supplement: Supplementary file 2 — Appendix S2 Detailed explanation of annual lifetime Health Utility Index Mark 3 (HUI3) calculations Appendix S3 References for supplementary materials Table S1 Data sources Table S2 Complete model parameters Table S3 Model outcomes for the base case Figure S1 One‐way deterministic sensitivity analysis. Figure S2 Two‐way deterministic sensitivity analysis. [file UOG-66-290-s001.docx]

**SUPPORTING INFORMATION**

**Appendix S1** Detailed calculations (Excel spreadsheet)

**Table S1** Data sources

**Table S2** Complete model parameters

**Appendix S**[**2**](#AS3) Detailed explanation of annual, lifetime Health Utility Index Mark 3 (HUI3) calculations

**Table S3** Model outcomes for the base case

**Figure S1** One-way deterministic sensitivity analysis

**Figure S2** Two-way deterministic sensitivity analysis

**Appendix S**[**3**](#AS4) References for supplementary materials

**Table S1.** Data sources. Evidence classification is based on the Canadian Task Force Classification of Study Design.^1^ Abbreviations: HRQoL, health-related quality of life; HUI3, health utility index mark 3; NICU, neonatal intensive care unit; NSI, neurosensory impairment.

| **Reference** | **Model parameters** | **Type of study** | **Level of evidence** | **Twin-specific** | **Canadian** |
| --- | --- | --- | --- | --- | --- |
| *Decision tree parameters* |  |  |  |  |  |
| Melamed AJOG 2016 (PMID 27365003) | Cervical length screening detection rates | Retrospective cohort | II-2 | Y | Y |
| Romero UOG 2022 (PMID: 34941003) | Progesterone effect on probability of birth timing | Meta-analysis | I | Y | N |
| Roman AJOG 2015 (PMID: 25637840) | Cerclage effect on probability of birth timing | Retrospective cohort | II-2 | Y | N |
| Melamed AJOG 2016 (PMID 27207277) | Probability of birth timing based on screening outcome | Retrospective cohort | II-2 | Y | Y |
| Braun Jama Netw Open 2020 (PMID: 32556257) | Probability of admission to NICU | Retrospect cohort (population) | II-2 | N | N |
| *cSTM parameters* |  |  |  |  |  |
| Cheong-See BMJ 2015 (PMID: 27599496) | Probability of stillbirth | Meta-analysis | II-2 | Y | N |
| Crump Lancet Child Adolesc Health 2019 (PMID: 30956154) | Annual risk of mortality due to prematurity | Retrospective cohort (population) | II-2 | N | N |
| Rios J Pediatrics 2021 (PMID: 32979384) | Inpatient NICU costs | Retrospective cohort | II-2 | N | Y |
| Johnston BMC Pediatrics 2014 (PMID: 24708755) | Annual health-related costs | Decision model | - | N | Y |
| Canadian community health survey (2015-2016) | Annual HRQoL (HUI3) | Cross-sectional | II-2 | N | Y |
| Saigal J Peds 2016 (PMID: 27592095) | HUI3 adjustment for prematurity and NSI | Prospective cohort | II-2 | N | Y |
| Vohr Pediatrics 2005 (PMID: 16143580) | Probability of NSI (<= 32+0 weeks) | Prospective cohort | II-2 | N | N |
| Johnson Arch Dis Child Fetal Neonatal Ed 2015 (PMID: 25834170) | Probability of NSI (> 32+0 weeks) | Prospective cohort (population0 | II-2 | N | N |

**Table S2.** Model parameters, their base case point estimates, clinical ranges (deterministic sensitivity analysis), probability distributions (probabilistic sensitivity analysis). Unless noted, clinical ranges represent 95% confidence intervals sourced either directly from publications or calculated using either a Wilson method for small sample sizes or a normal approximation for large sample sizes (see **Appendix S1** for full calculations).

| **MODEL PARAMETER** | **BASE CASE** | **CLINICAL RANGE** | **PROBABILIY DISTRIBUTION†** | **REFERENCES/COMMENTS** |
| --- | --- | --- | --- | --- |
| **1. DECISION TREE PROBABILITIES** | | | | |
| Cervical length screening detection | | | | |
| Screen one CL ≤ 25 mm | 0.044 | 0.03, 0.06 | beta(32, 696) | Melamed AJOG 2016 (PMID 27365003)^2^ |
| Screen one CL ≤ 15 mm, given CL ≤ 25 | 0.468 | 0.31, 0.64 | beta(15, 17) | Melamed AJOG 2016 (PMID 27365003)^2^ |
| Screen two CL ≤ 25 mm, given screen one negative (> 25 mm) | 0.076 | 0.06, 0.10 | beta(53, 643) | Melamed AJOG 2016 (PMID 27365003)^2^ |
| Screen two CL ≤ 15 mm, given CL ≤ 25 and screen one negative | 0.377 | 0.26, 0.51 | beta(20, 33) | Melamed AJOG 2016 (PMID 27365003)^2^ |
| Treatment uptake (%) | | | | |
| Progesterone uptake when CL ≤ 25 mm | 1 | 0.5, 1 | - | Assumption |
| Cerclage uptake when CL ≤ 15 mm and taking progesterone | 1 | 0, 1 | - | Assumption |
| Cerclage uptake when CL ≤ 15 mm and NOT taking progesterone | 0 | 0, 0.5 | - | Assumption |
| Progesterone effect (relative risk) on probability of birth at | | | | |
| ≤ 28+0 weeks | 0.53 | 0.24, 1.16 | lognormal(log(0.53), 0.40) | Romero UOG 2022 (PMID: 34941003)^3^ |
| 28+1**-**32+0 weeks | 0.71 | 0.34, 1.47 | lognormal(log(0.71), 0.37) | Romero UOG 2022 (PMID: 34941003)^3^ |
| 32+1**-**34+0 weeks | 0.64 | 0.31, 1.33 | lognormal(log(0.64), 0.37) | Romero UOG 2022 (PMID: 34941003)^3^ |
| 34+1**-**37+0 weeks | 1.02 | 0.72, 1.43 | lognormal(log(1.02), 0.17) | Romero UOG 2022 (PMID: 34941003)^3^ |
| Cerclage effect* (relative risk) on probability of birth at | | | | |
| ≤ 28+0 weeks | 0.56 | 0.26, 1.21 | lognormal(log(0.56), 0.39) | Roman AJOG 2015 (PMID: 25637840)^4^ |
| 28+1**-**32+0 weeks | 0.78 | 0.38, 1.60 | lognormal(log(0.78), 0.37) | Roman AJOG 2015 (PMID: 25637840)^4^ |
| 32+1**-**34+0 weeks | 0.3 | 0.11, 0.84 | lognormal(log(0.30), 0.53) | Roman AJOG 2015 (PMID: 25637840)^4^ |
| 34+1**-**37+0 weeks | 1 | 0.60, 1.67 | lognormal(log(1.00), 0.26) | Roman AJOG 2015 (PMID: 25637840)^4^ |
| Probability of birth timing based on screen outcome | | | | |
| Screening negative (CL ≥ 25 mm x2) | | | | |
| ≤ 28+0 weeks | 0.011 | 0.01, 0.02 | beta(7, 642) | Melamed AJOG 2016 (PMID 27207277)^5^ |
| 28+1**-**32+0 weeks | 0.026 | 0.02, 0.04 | beta(17, 625) | Melamed AJOG 2016 (PMID 27207277)^5^ |
| 32+1**-**34+0 weeks | 0.062 | 0.05, 0.08 | beta(39, 586) | Melamed AJOG 2016 (PMID 27207277)^5^ |
| 34+1**-**37+0 weeks | 0.573 | 0.53, 0.61 | beta(336, 250) | Melamed AJOG 2016 (PMID 27207277)^5^ |
| Screen one positive (CL 16-25 mm) | | | | |
| ≤ 28+0 weeks | 0.118 | 0.03, 0.34 | beta(2, 15) | Melamed AJOG 2016 (PMID 27207277)^5^ |
| 28+1**-**32+0 weeks | 0.133 | 0.04, 0.38 | beta(2, 13) | Melamed AJOG 2016 (PMID 27207277)^5^ |
| 32+1**-**34+0 weeks | 0.077 | 0.01, 0.33 | beta(1, 12) | Melamed AJOG 2016 (PMID 27207277)^5^ |
| 34+1**-**37+0 weeks | 0.5 | 0.25, 0.75 | beta(6, 6) | Melamed AJOG 2016 (PMID 27207277)^5^ |
| Screen one positive (CL ≤ 15 mm) | | | | |
| ≤ 28+0 weeks | 0.533 | 0.30, 0.75 | beta(8, 7) | Melamed AJOG 2016 (PMID 27207277)^5^ |
| 28+1**-**32+0 weeks | 0.143 | 0.03, 0.51 | beta(1, 7) | Melamed AJOG 2016 (PMID 27207277)^5^ |
| 32+1**-**34+0 weeks | 0 | 0.00, 0.39 | beta(0, 6) | Melamed AJOG 2016 (PMID 27207277)^5^ |
| 34+1**-**37+0 weeks | 0.5 | 0.19, 0.81 | beta(3, 6) | Melamed AJOG 2016 (PMID 27207277)^5^ |
| Screen two positive (CL 16-25 mm) | | | | |
| ≤ 28+0 weeks | 0.087 | 0.03, 0.2 | beta(4, 42) | Melamed AJOG 2016 (PMID 27207277)^5^ |
| 28+1**-**32+0 weeks | 0.119 | 0.05, 0.25 | beta(5, 37) | Melamed AJOG 2016 (PMID 27207277)^5^ |
| 32+1**-**34+0 weeks | 0.135 | 0.06, 0.28 | beta(5, 32) | Melamed AJOG 2016 (PMID 27207277)^5^ |
| 34+1**-**37+0 weeks | 0.563 | 0.39, 0.72 | beta(18, 14) | Melamed AJOG 2016 (PMID 27207277)^5^ |
| Screen two positive (CL ≤ 15 mm) | | | | |
| ≤ 28+0 weeks | 0.303 | 0.17, 0.47 | beta(10, 23) | Melamed AJOG 2016 (PMID 27207277)^5^ |
| 28+1**-**32+0 weeks | 0.304 | 0.16, 0.51 | beta(7, 16) | Melamed AJOG 2016 (PMID 27207277)^5^ |
| 32+1**-**34+0 weeks | 0.063 | 0.01, 0.28 | beta(1, 15) | Melamed AJOG 2016 (PMID 27207277)^5^ |
| 34+1**-**37+0 weeks | 0.533 | 0.3, 0.75 | beta(8, 7) | Melamed AJOG 2016 (PMID 27207277)^5^ |
| Admission to NICU | | | | |
| ≤ 28+0 weeks | 1 | - | - | Assumption |
| 28+1**-**32+0 weeks | 1 | - | - | Assumption |
| 32+1**-**34+0 weeks | 1 | - | - | Assumption |
| 34+1**-**37+0 weeks | 0.46 | 0.25, 1.0 | beta(10280, 12016) | Braun Jama Netw Open 2020 (PMID: 32556257).^6^ Range: min, max. |
| > 37+0 weeks | 0.07 | 0.07, 0.09 | beta(19270, 244226) | Braun Jama Netw Open 2020 (PMID: 32556257).^6^ Range: min, max. |
| 2. cSTM PROBABILITIES | | | | |
| Stillbirth | | | | |
| ≤ 28+0 weeks | 0.0007 | 0.0004, 0.0011 | beta(20, 26665) | Cheong-See BMJ 2015 (PMID: 27599496)^7^ |
| 28+1**-**32+0 weeks | 0.0015 | 0.0012, 0.0018 | beta(78, 51680) | Cheong-See BMJ 2015 (PMID: 27599496)^7^ |
| 32+1**-**34+0 weeks | 0.002 | 0.0015, 0.0026 | beta(49, 24129) | Cheong-See BMJ 2015 (PMID: 27599496)^7^ |
| 34+1**-**37+0 weeks | 0.0011 | 0.0008, 0.0014 | beta(51, 45073) | Cheong-See BMJ 2015 (PMID: 27599496)^7^ |
| > 37+0 weeks | 0.0057 | 0.0042, 0.0071 | beta(58, 10151) | Cheong-See BMJ 2015 (PMID: 27599496)^7^ |
| Annual mortality rate | | | | |
| Age-dependent mortality in Canada | Multiple | - | - | Human Mortality Database (2020)^8^ |
| Hazard ratio (0 to <1 years) | | | | |
| ≤ 28+0 weeks | 236.32 | 223.81, 249.54 | lognormal(log(236.32), 0.03) | Crump Lancet Child Adolesc Health 2019 (PMID: 30956154)^9^ |
| 28+1**-**32+0 weeks | 32.1 | 30.49, 33.79 | lognormal(log(32.10), 0.03) | Crump Lancet Child Adolesc Health 2019 (PMID: 30956154)^9^ |
| 32+1**-**34+0 weeks | 32.1 | 30.49, 33.79 | lognormal(log(32.10), 0.03) | Crump Lancet Child Adolesc Health 2019 (PMID: 30956154)^9^ |
| 34+1**-**37+0 weeks | 6.75 | 6.39, 7.13 | lognormal(log(6.75), 0.03) | Crump Lancet Child Adolesc Health 2019 (PMID: 30956154)^9^ |
| > 37+0 weeks | 1 | - | - | Reference value |
| Hazard ratios (1-9 years) | | | | |
| ≤ 28+0 weeks | 4.52 | 3.02, 6.75 | lognormal(log(4.52), 0.21) | Crump Lancet Child Adolesc Health 2019 (PMID: 30956154)^9^ |
| 28+1**-**32+0 weeks | 3.26 | 2.78, 3.82 | lognormal(log(3.26), 0.08) | Crump Lancet Child Adolesc Health 2019 (PMID: 30956154)^9^ |
| 32+1**-**34+0 weeks | 3.26 | 2.78, 3.82 | lognormal(log(3.26), 0.08) | Crump Lancet Child Adolesc Health 2019 (PMID: 30956154)^9^ |
| 34+1**-**37+0 weeks | 1.9 | 1.7, 2.11 | lognormal(log(1.9), 0.05) | Crump Lancet Child Adolesc Health 2019 (PMID: 30956154)^9^ |
| > 37+0 weeks | 1 | - | - | Reference value |
| Hazard ratios (10-19 years) | | | | |
| ≤ 28+0 weeks | 1.89 | 0.98, 3.63 | lognormal(log(1.89), 0.33) | Crump Lancet Child Adolesc Health 2019 (PMID: 30956154)^9^ |
| 28+1**-**32+0 weeks | 1.91 | 1.56, 2.33 | lognormal(log(1.91), 0.10) | Crump Lancet Child Adolesc Health 2019 (PMID: 30956154)^9^ |
| 32+1**-**34+0 weeks | 1.91 | 1.56, 2.33 | lognormal(log(1.91), 0.10) | Crump Lancet Child Adolesc Health 2019 (PMID: 30956154)^9^ |
| 34+1**-**37+0 weeks | 1.37 | 1.21, 1.55 | lognormal(log(1.37), 0.06) | Crump Lancet Child Adolesc Health 2019 (PMID: 30956154)^9^ |
| > 37+0 weeks | 1 | - |  | Reference value |
| Hazard ratios (20-29 years) | | | | |
| ≤ 28+0 weeks | 2.12 | 1.26, 3.59 | lognormal(log(2.12), 0.27) | Crump Lancet Child Adolesc Health 2019 (PMID: 30956154)^9^ |
| 28+1**-**32+0 weeks | 1.42 | 1.19, 1.7 | lognormal(log(1.42), 0.09) | Crump Lancet Child Adolesc Health 2019 (PMID: 30956154)^9^ |
| 32+1**-**34+0 weeks | 1.42 | 1.19, 1.7 | lognormal(log(1.42), 0.09) | Crump Lancet Child Adolesc Health 2019 (PMID: 30956154)^9^ |
| 34+1**-**37+0 weeks | 1.35 | 1.23, 1.48 | lognormal(log(1.35), 0.05) | Crump Lancet Child Adolesc Health 2019 (PMID: 30956154)^9^ |
| > 37+0 weeks | 1 | - | - | Reference value |
| Hazard ratios (30-45 years) | | | | |
| ≤ 28+0 weeks | 2.04 | 0.92, 4.55 | lognormal(log(2.04), 0.41) | Crump Lancet Child Adolesc Health 2019 (PMID: 30956154)^9^ |
| 28+1**-**32+0 weeks | 1.48 | 1.17, 1.87 | lognormal(log(1.48), 0.12) | Crump Lancet Child Adolesc Health 2019 (PMID: 30956154)^9^ |
| 32+1**-**34+0 weeks | 1.48 | 1.17, 1.87 | lognormal(log(1.48), 0.12) | Crump Lancet Child Adolesc Health 2019 (PMID: 30956154)^9^ |
| 34+1**-**37+0 weeks | 1.22 | 1.07, 1.39 | lognormal(log(1.22), 0.07) | Crump Lancet Child Adolesc Health 2019 (PMID: 30956154)^9^ |
| > 37+0 weeks | 1 | - | - | Reference value |
| **3. MODEL COSTS (2023 CAD$)** | | | | |
| One ultrasound scan | 75.3 | 50, 566 | - | Ontario schedule of benefits 2023 for base case.^10^ Maximum range from Werner Ultrasound Obstet Gynecol 2011 (PMID: 21157771).^11^ |
| Cerclage administration | 3,614.36 | 2400, 4374 | - | CIHI 2023^12^ |
| Progesterone prescription per week | 20.16 | 15, 25 | - | Ontario Formulary 2023^13^ |
| Deliver cost | 5,489 | 2,596, 12,677 | - | CIHI 2023^12^, Gu J Obstet Gynaecol Can 2020 (PMID: 3188375).^14^ Range: min, max costs in Canada. |
| Initial inpatient (NICU) costs | | | | |
| ≤ 28+0 weeks | 121,650 | 2,781, 633,131 | gamma(m=121,650, SE=2,211) | Rios J Pediatrics 2021 (PMID: 32979384).^15^ Range: min, max. |
| 28+1**-**32+0 weeks | 42,678 | 1,949, 593,043 | gamma(m=42,678, SE=779) | Rios J Pediatrics 2021 (PMID: 32979384).^15^ Range: min, max. |
| 32+1**-**34+0 weeks | 28,034 | 1,506, 351,495 | gamma(m=28,034, SE=553) | Rios J Pediatrics 2021 (PMID: 32979384).^15^ Range: min, max. |
| 34+1**-**37+0 weeks | 17,243 | 1,506, 377,443 | gamma(m=17,243, SE=413) | Rios J Pediatrics 2021 (PMID: 32979384).^15^ Range: min, max. |
| > 37+0 weeks | 17,243 | 1,506, 377,443 | gamma(m=17,243, SE=413) | Assumed equal to 36 week estimates |
| Annual costs (cSTM) | | | | |
| Alive state by GA category | Multiple | Multiple | gamma(m, SD) | Johnston BMC Pediatrics 2014 (PMID: 24708755)^16^ |
| Dead state | 0 | - | - | Assumption |
| Annual discounting | 0.015 | 0.00, 0.03 | 0 | CADTH 2017^17^ |
| **4. MODEL UTILITIES** | | | | |
| HRQoL parameters | | | | |
| HUI3 values | | | | |
| Age 0, at term | 0.871 | 0.826, 0.916 | beta(m=0.871, SE=0.023) | Saigal J Peds 2016 (PMID: 27592095)^18^ |
| Change from age 0 to 100 | -0.211 | -0.297, -0.125 | beta(m=-0.211, SE = 0.044) | Canadian community health survey (2015-2016)^19^ |
| Prematurity and NSI effect | -0.264 | -0.358, -0.170 | normal(-0.264, 0.048) | Saigal J Peds 2016 (PMID: 27592095)^18^ |
| Prematurity effect (≤ 28+0 weeks) | -0.092 | -0.157, -0.027 | normal(-0.092, 0.033) | Saigal J Peds 2016 (PMID: 27592095)^18^ |
| Prematurity effect (28+1-32+0 weeks) | 0 | -0.100, 0.000 | - | Assumption |
| Prematurity effect (32+1**-**34+0 weeks) | 0 | -0.100, 0.000 | - | Assumption |
| Prematurity effect (34+1**-**37+0 weeks) | 0 | - | - | Assumption (reference group) |
| Probability of neuro-sensory impairment | | | | |
| ≤ 28+0 weeks | 0.45 | 0.41, 0.48 | beta(406, 504) | Vohr Pediatrics 2005 (PMID: 16143580)^20^ |
| 28+1**-**32+0 weeks | 0.28 | 0.24, 0.32 | beta(142, 370) | Vohr Pediatrics 2005 (PMID: 16143580)^20^ |
| 32+1**-**34+0 weeks | 0.05 | 0.00, 0.09 | beta(4, 83) | Johnson Arch Dis Child Fetal Neonatal Ed 2015 (PMID: 25834170)^21^ |
| 34+1**-**37+0 weeks | 0.07 | 0.05, 0.09 | beta(40, 511) | Johnson Arch Dis Child Fetal Neonatal Ed 2015 (PMID: 25834170)^21^ |
| > 37+0 weeks | 0.02 | 0.01, 0.04 | beta(19, 746) | Johnson Arch Dis Child Fetal Neonatal Ed 2015 (PMID: 25834170) |
| Other utilities | | | | |
| Dead state | 0 | - | - | Assumption |
| Annual discounting | 0.015 | 0.00, 0.03 | - | CADTH 2017^17^ |

*The effect of cerclage effect in combination with progesterone is not known and assumed conservatively to be the same as effect of cerclage alone.

† Distributions: beta (number of events, number of non-events); lognormal(log(mean), standard error); gamma (mean, standard error), normal (mean, standard error).

Abbreviations: CL, cervical length; cSTM, cohort state transition model; GA, gestational age; HRQoL, health-related quality of life; HUI3, Health Utility Index Mark 3; NICU, neonatal intensive care unit.

**Appendix S2.** Detailed explanation of annual, lifetime Health Utility Index Mark 3 (HUI3) calculations. Abbreviations: CL, cervical length. GA, gestational age; NSI, neuro-sensory impairment.

Annual HRQoL values were calculated for each GA category from 0 to 100 years using several steps. First, a population trend was identified via Health Utility Index Mark 3 (HUI3) data collected in the latest available Canadian Community Health Survey (CCHS, 2015-2016, n=2,769).^19^ HUI3 estimates were available from age 12 to 80+. Because of a paucity of long-term data for premature birth, HRQoL estimates were sourced from a Canadian population born with extremely low birth weight (< 1000 g).^18^ HUI3 data were collected for this group (n=153) during three time-periods, the latest at 29-36 years. Linear trajectories using multilevel modeling were reported for patients with and without neuro-sensory impairment (NSI). The average sample GA at delivery was 26-27 weeks, and these results are used to approximate the HRQoL of the ≤ 28 weeks GA group in our model. This comparison is further supported by previous work demonstrating the median birthweight among twins born at 27 weeks to be 1000 g.^22^ Controls in the Canadian low birth weight study were term born with birth weight > 2500 g (n=137). Notably, these results are not specific to twins.

Annual HRQoL curves for each GA category were calculated as follows (see figure below of baseline curves). First, a function was determined for patients born at term. This function assumed that HUI3 at age 0 (i.e. the y intercept) was equal to that reported in the term-born controls;^18^ change in HUI3 values from age 0 to 100 was derived from the CCHS.^23^ The function has the general form derived based on the best visual fit of the data: y = HUI3_100_+ (HUI3_0_ - HUI3_100_)/[1+ (age/70)^8.4^]. Next, for each GA category, two curves were created: one for prematurity without neurosensory impairment (NSI) and one for prematurity with NSI. The curves were shifted along the y axis based on the y intercept values reported by Saigal^18^ for these two subgroups. The impact of prematurity without NSI on GA greater than 28 weeks is not known, and was thus assumed conservatively to have no impact. The y-axis shift for the premature with NSI group was kept constant for all GA categories, assuming that the impact of NSI on HUI3 is similar, regardless of GA at delivery. Finally, a single curve was assigned for each GA category by taking a weighted average of the two curves (premature without NSI and premature with NSI), based on the proportion of births resulting in NSI. Proportion of NSI for each GA category were sourced from two observational studies.^20,21^ This approach does not account for the effect of a different mortality rate in the NSI group on the overall HUI3 averages. Additionally, while the data generated by Saigal^18^ is linear, the model function is non-linear and likely overestimates HUI3 values for extreme prematurity (≤ 28 weeks), biasing against the CL screening strategies.

.

Health Utility Index Mark 3 (HUI3) life-time estimates for the base case. The dotted line represents weighted HUI3 estimates from the Canadian Community Health Survey (2015-2016). Abbreviations: GA, gestational age.

**Table S3.** Base case outcomes. Outcomes regarding twins are reported for both twins, assuming identical outcomes. Abbreviations: NICU, neonatal intensive care unit, QALY, quality-adjusted life years.

| **Outcome** | **No screen** | **One-step** | **Two-step** |
| --- | --- | --- | --- |
| Proportion screen positive |  |  |  |
| ≤ 15 mm | 0 | 0.02 | 0.05 |
| ≤ 25 mm | 0 | 0.04 | 0.12 |
| Proportion preterm |  |  |  |
| ≤ 28+0 weeks | 0.04 | 0.03 | 0.02 |
| ≤ 32+0 weeks | 0.07 | 0.07 | 0.06 |
| ≤ 34+0 weeks | 0.13 | 0.13 | 0.12 |
| Stillbirths/1000 pregnancies | 5.74 | 5.77 | 5.81 |
| Life expectancy, years | 154.67 | 155.31 | 155.93 |
| Cost per pregnancy |  |  |  |
| Ultrasound | $0 | $95 | $194 |
| Progesterone | $0 | $14 | $39 |
| Cerclage | $0 | $74 | $173 |
| NICU admission | $23,954 | $22,507 | $21,093 |
| Total costs | $46,024 | $44,682 | $43,407 |
| Total QALYs | 74.99 | 75.33 | 75.66 |

**Figure S1.** Results of the deterministic, one-way sensitivity analysis for model costs (a), quality-adjusted life years (QALYs) (b), preterm birth before 28+0 weeks (c), and preterm birth before 34+0 weeks (d). Each plot represents a single outcome and the dominant strategy for that outcome (i.e. lowest cost, highest QALYs, or lowest proportion of preterm birth). The parameter values are standardized to a common scale (0-1) to allow for simpler visualization. The (+) symbol represents the base case for each variable. Abbreviations: C, cerclage; CL, cervical length; HR, hazard ratio; P, progesterone.

*Begins on next page*

**Figure S2.** Results of the deterministic, two-way sensitivity analysis for model total costs. (a) Progesterone cost vs. effectiveness, and (b) cerclage effectiveness vs. cerclage costs. Cerclage is assumed to be done concurrently with progesterone therapy, but the effectiveness of cerclage is sourced from data on cerclage alone. Each plot shows the strategy with the lowest total model cost across ranges of two parameters. Base case values are represented by a (+) sign.

a. Progesterone effectiveness vs. progesterone cost

b. Cerclage effectiveness vs. cerclage cost

**Appendix S3.** References for supplementary materials

1. Tulandi T, Balayla J. Study Designs and the Use of the Canadian Task Force Classification. *J Obstet Gynaecol Can*. 2018;40(11):1383-1384. doi:10.1016/J.JOGC.2018.08.011

2. Melamed N, Pittini A, Hiersch L, et al. Do serial measurements of cervical length improve the prediction of preterm birth in asymptomatic women with twin gestations? *Am J Obstet Gynecol*. 2016;215(5):616.e1-616.e14. doi:10.1016/J.AJOG.2016.06.034

3. Romero R, Conde-Agudelo A, Rehal A, et al. Vaginal progesterone for the prevention of preterm birth and adverse perinatal outcomes in twin gestations with a short cervix: an updated individual patient data meta-analysis. *Ultrasound Obstet Gynecol*. 2022;59(2):263-266. doi:10.1002/uog.24839

4. Roman A, Rochelson B, Fox NS, et al. Efficacy of ultrasound-indicated cerclage in twin pregnancies. *Am J Obstet Gynecol*. 2015;212(6):788.e1-788.e6. doi:10.1016/J.AJOG.2015.01.031

5. Melamed N, Pittini A, Hiersch L, et al. Serial Cervical Length Determination in Twin Pregnancies Reveals Four Distinct Patterns with Prognostic Significance for Preterm Birth. *Am J Obstet Gynecol*. 2016;215(4):476.e1. doi:10.1016/J.AJOG.2016.05.018

6. Braun D, Braun E, Chiu V, et al. Trends in Neonatal Intensive Care Unit Utilization in a Large Integrated Health Care System. *JAMA Netw Open*. 2020;3(6). doi:10.1001/JAMANETWORKOPEN.2020.5239

7. Cheong-See F, Schuit E, Arroyo-Manzano D, et al. Prospective risk of stillbirth and neonatal complications in twin pregnancies: systematic review and meta-analysis. *BMJ*. 2016;354. doi:10.1136/BMJ.I4353

8. Human Mortality Database: Canada. Accessed December 13, 2023. https://www.mortality.org/Country/Country?cntr=CAN

9. Crump C, Sundquist J, Winkleby MA, Sundquist K. Gestational age at birth and mortality from infancy into mid-adulthood: a national cohort study. *Lancet Child Adolesc Health*. 2019;3(6):408-417. doi:10.1016/S2352-4642(19)30108-7

10. Ontario Schedule of Benefits: Physician Services Under the Health Insurance Act. 2023. Accessed June 11, 2024. https://www.ontario.ca/files/2024-01/moh-ohip-schedule-of-benefits-2024-01-24.pdf

11. Werner EF, Hamel MS, Orzechowski K, Berghella V, Thung SF. Cost-effectiveness of transvaginal ultrasound cervical length screening in singletons without a prior preterm birth: an update. *Am J Obstet Gynecol*. 2015;213(4):554.e1-554.e6. doi:10.1016/J.AJOG.2015.06.020

12. Patient Cost Estimator | CIHI. Accessed December 12, 2023. https://www.cihi.ca/en/patient-cost-estimator

13. Formulary Search - Search Results. Accessed December 12, 2023. https://www.formulary.health.gov.on.ca/formulary/results.xhtml?q=progesterone&type=1

14. Gu J, Karmakar-Hore S, Hogan ME, et al. Examining Cesarean Section Rates in Canada Using the Modified Robson Classification. *Journal of Obstetrics and Gynaecology Canada*. 2020;42(6):757-765. doi:10.1016/J.JOGC.2019.09.009

15. Rios JD, Shah PS, Beltempo M, et al. Costs of Neonatal Intensive Care for Canadian Infants with Preterm Birth. *J Pediatr*. 2021;229:161-167.e12. doi:10.1016/J.JPEDS.2020.09.045

16. Johnston KM, Gooch K, Korol E, et al. The economic burden of prematurity in Canada. *BMC Pediatr*. 2014;14(1). doi:10.1186/1471-2431-14-93

17. Guidelines for the Economic Evaluation of Health Technologies: Canada — 4th Edition | CADTH. Accessed December 13, 2023. https://www.cadth.ca/guidelines-economic-evaluation-health-technologies-canada-4th-edition

18. Saigal S, Ferro MA, Van Lieshout RJ, Schmidt LA, Morrison KM, Boyle MH. Health-Related Quality of Life Trajectories of Extremely Low Birth Weight Survivors into Adulthood. *J Pediatr*. 2016;179:68-73.e1. doi:10.1016/j.jpeds.2016.08.018

19. Division HS. Canadian Community Health Survey, 2015-2016: Annual Component. Published online October 23, 2023. doi:10.5683/SP3/YJBSEJ

20. Vohr BR, Wright LL, Poole WK, McDonald SA. Neurodevelopmental outcomes of extremely low birth weight infants. *Pediatrics*. 2005;116(3):635-643. doi:10.1542/PEDS.2004-2247

21. Johnson S, Evans TA, Draper ES, et al. Neurodevelopmental outcomes following late and moderate prematurity: a population-based cohort study. *Arch Dis Child Fetal Neonatal Ed*. 2015;100(4):F301-8. doi:10.1136/archdischild-2014-307684

22. Arbuckle TE, Wilkins R, Sherman GJ. Birth weight percentiles by gestational age in Canada. *Obstetrics and gynecology*. 1993;81(1):39-48. http://www.ncbi.nlm.nih.gov/pubmed/8416459

23. Health Statistics Division. Canadian Community Health Survey, 2015-2016: Annual Component.
